# LMNA-NTRK1-rearranged spindle cell neoplasm with multiple relapses: a case report and literature review

**DOI:** 10.3389/fmed.2025.1691619

**Published:** 2025-12-02

**Authors:** Yinan Zhu, Ziyue Wang, Haiyan Xi, Wanchen Lu, Mingfang Sun, Xuyong Lin

**Affiliations:** Department of Pathology, The First Affiliated Hospital and College of Basic Medical Sciences, China Medical University, Shenyang, China

**Keywords:** NTRK fusion, spindle cell neoplasm, LMNA::NTRK1, malignant transformation, recurrence, pigmentation

## Abstract

Neurotrophic tyrosine receptor kinase (NTRK)-rearranged spindle cell neoplasms (NTRK-RSCNs) constitute a rare, heterogeneous subset of soft tissue tumors defined by oncogenic fusions involving NTRK1, NTRK2, or NTRK3 genes. Despite the remarkable efficacy of TRK inhibitor therapy in fusion-positive tumors, the histomorphologic variability of NTRK-RSCNs poses significant diagnostic challenges, and data on malignant transformation remain limited. Herein, we report a unique case of LMNA::NTRK1-rearranged spindle cell neoplasm in a 23-year-old woman, characterized by previously undescribed pigmentation, multiple local recurrences, and fibrosarcoma-like malignant transformation—features that have not been documented in prior literature. Through integrated histopathological, immunohistochemical, and molecular analyses, we characterize the diagnostic nuances, biological behavior, and potential drivers of progression in this entity. Our findings expand the morphological and clinical spectrum of LMNA::NTRK1-rearranged tumors and highlight the need for close follow-up and consideration of adjuvant targeted therapy in high-risk cases.

## 1 Introduction

Neurotrophic tyrosine receptor kinase (NTRK)-rearranged spindle cell neoplasms (NTRK-RSCNs) are a recently recognized category of mesenchymal tumors, formally classified in the 2020 WHO Classification of Soft Tissue and Bone Tumors as distinct entities based on their molecular signature and therapeutic relevance (1). These tumors are driven by oncogenic fusions involving NTRK genes (NTRK1, NTRK2, NTRK3), which encode tropomyosin receptor kinase (TRK) proteins—transmembrane receptors critical for cell proliferation, differentiation, and survival (2). Among NTRK-RSCN subtypes, LMNA::NTRK1 fusion represents an emerging variant, with only 39 cases reported in the literature to date (2016–2025) (3–27).

LMNA::NTRK1-rearranged tumors exhibit a broad age distribution (0.2–57 years; median 19 years), with slightly more cases occurring in female individuals (52.5%), and a predilection for the limbs/trunk (62.5%), followed by the head/neck (17.5%) and gastrointestinal tract (15%) (3–27). Histopathologically, they typically present as hypocellular spindle cell proliferations in a collagenous stroma (fibromatosis-like), with fascicular growth or lymphoplasmacytic infiltration (inflammatory myofibroblastic tumor-like) and consistent immunophenotypic features including CD34, S100, and Pan-TRK positivity (3, 7, 12). Clinically, the majority of cases follow an indolent course, with an 8.8% local recurrence rate and a 2.9% distant metastasis rate after surgical resection (3–27). However, rare morphological variants (e.g., pigmented lesions) and malignant transformation have not been previously documented, creating gaps in our understanding of the biological spectrum and prognostic factors of these tumors.

Accurate diagnosis of LMNA::NTRK1-rearranged neoplasms is clinically critical due to the availability of TRK inhibitors, which achieve response rates exceeding 75% in NTRK fusion-positive solid tumors (2, 28). Nevertheless, diagnostic challenges persist due to histomorphologic overlap with mimickers such as desmoid-type fibromatosis, solitary fibrous tumor (SFT), and gastrointestinal stromal tumor (GIST) (3, 15). Furthermore, the factors driving recurrence and malignant transformation in these tumors remain unclear, with no prior reports linking p53 mutation to disease progression.

To address these knowledge gaps, we present the first case of LMNA::NTRK1-rearranged spindle cell neoplasm with pigmentation, multiple local recurrences, and fibrosarcoma-like malignant transformation. We discuss the diagnostic utility of integrated histopathological and molecular testing, the potential role of p53 mutation in tumor progression, and the clinical implications for adjuvant therapy.

## 2 Case presentation

### 2.1 Clinical information

A 23-year-old Asian woman presented to our institution with a painless scalp nodule that had been present for a duration of 10 years. Physical examination revealed a slightly elevated lesion measuring 1.0 cm × 0.8 cm on the right parietal scalp, with normal overlying skin and no tenderness or lymphadenopathy. The lesion had remained stable in size until 21 months prior to presentation, when the patient noted gradual enlargement. No relevant personal or family history of tumors was identified.

### 2.2 Pathological findings

#### 2.2.1 Gross pathology

The resected specimen was a 2.0 cm × 1.8 cm × 1.5 cm firm nodule with a grayish-white cut surface, well-circumscribed margins, and no evidence of necrosis. The overlying skin contained intact hair follicles.

#### 2.2.2 Histopathology

**Figure 1:**
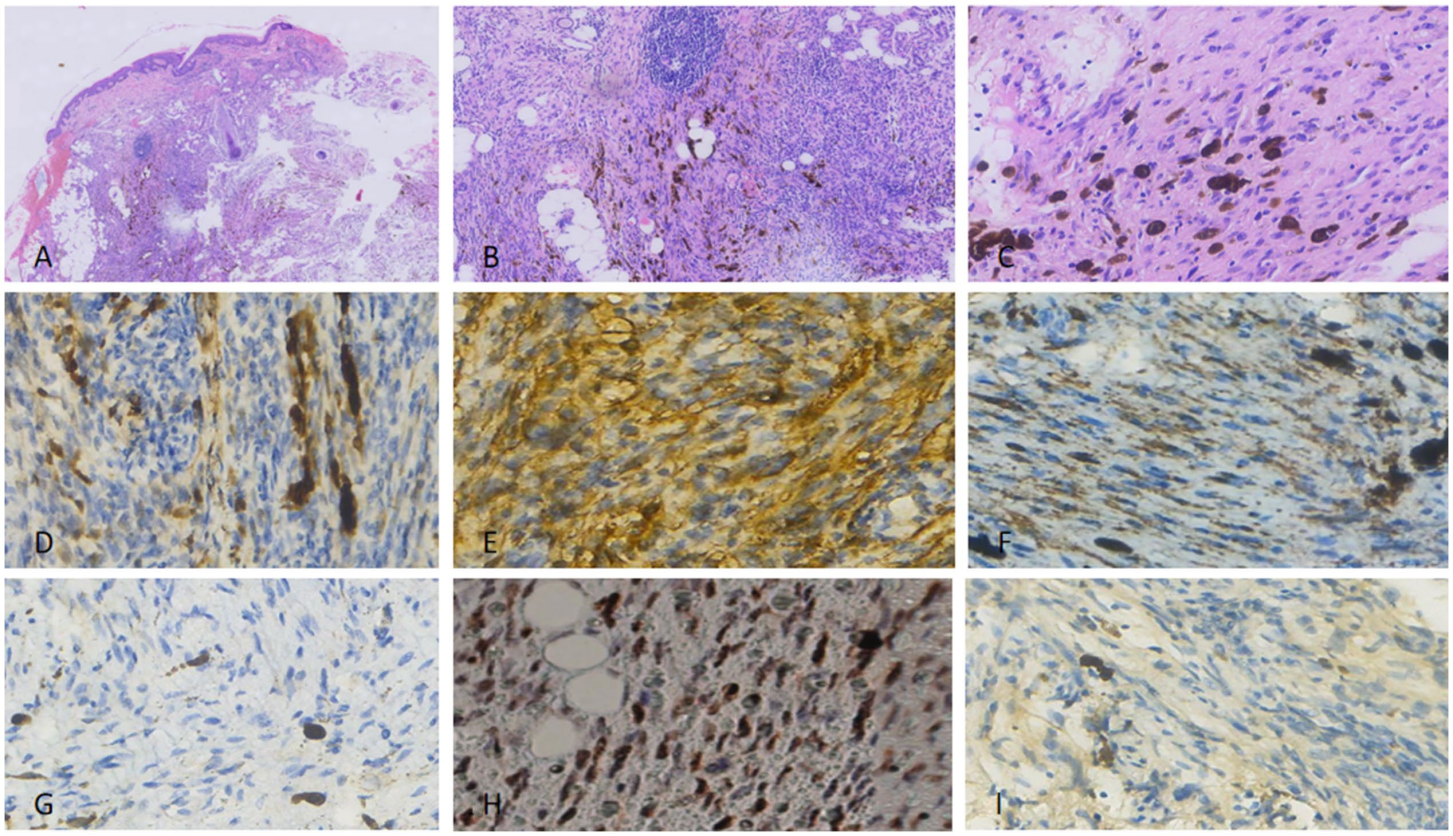
For the excised specimen from the first surgery, fusiform cells of uniform size were arranged, with fat visible in the interstitium **(A)**. The surrounding area showed lymphocyte aggregation **(B)** and pigment cell deposition **(C)**. Nuclear atypia and pathological mitoses were rare. Immunohistochemically, the tumor cells were focally positive for S100 **(D)**, while diffusely positive for CD34 **(E)** and P-TRK **(F)**. SMA **(G)** was completely negative. P53 expression was diffusely positive, consistent with overexpression related to mutation **(H)**. Ki-67 proliferation index was not more than 2% **(I)**.

Tumor cells were spindle-shaped with uniform morphology, arranged in a matted pattern—defined as irregularly clustered, merging nodules with ill-defined inter-nodular boundaries (distinct from fascicular or storiform architectures) (Figure 1A). Prominent pigment deposition, spindle cell proliferation, and lymphocytic infiltration were observed (Figure 1B). Scattered dendritic pigment-containing cells were identified in the stroma (Figure 1C). Tumor cells exhibited mild nuclear polymorphism with rare mitotic figures (<1/10 high-power fields [HPF]). Lymphocytes, some forming germinal centers, were distributed between spindle cells and adipocytes.

#### 2.2.3 Immunohistochemistry (IHC)

**Figure 2:**
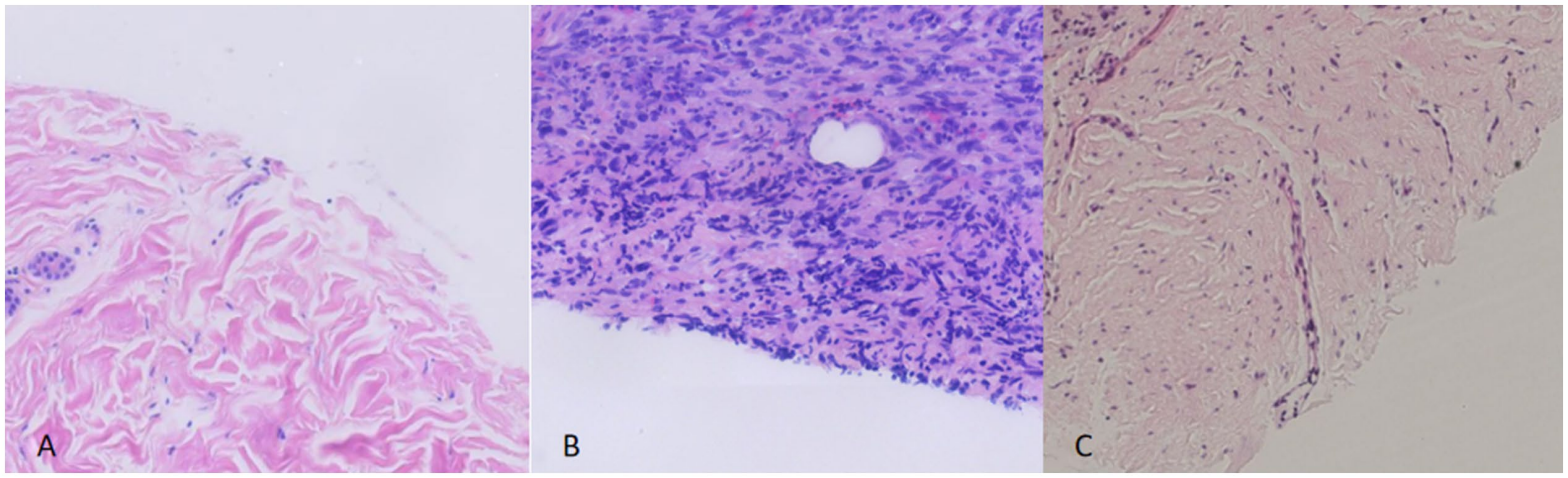
In the first **(A)** and third surgeries **(C)**, no tumor cells were detected at the incision margin on pathological examination, whereas tumor cells were identified at the incision margin in the second surgery **(B)**.

Tumor cells were positive for *β*-catenin (diffuse), vimentin (diffuse), S100 (90% of cells; Figure 1D), CD34 (80% of cells; Figure 1E), Bcl-2 (focal), Pan-TRK (diffuse; Figure 1F), and CD99 (focal). They were negative for SMA (Figure 1G), TLE-1, Melan A, EMA, STAT6, Sox10, and Desmin. Diffuse nuclear positivity for p53 was detected (consistent with pathogenic mutation; Figure 1H). The Ki-67 proliferation index was <2% (Figure 1I). Surgical margins were negative for tumor (Figure 2A).

#### 2.2.4 Molecular testing

**Figure 3:**
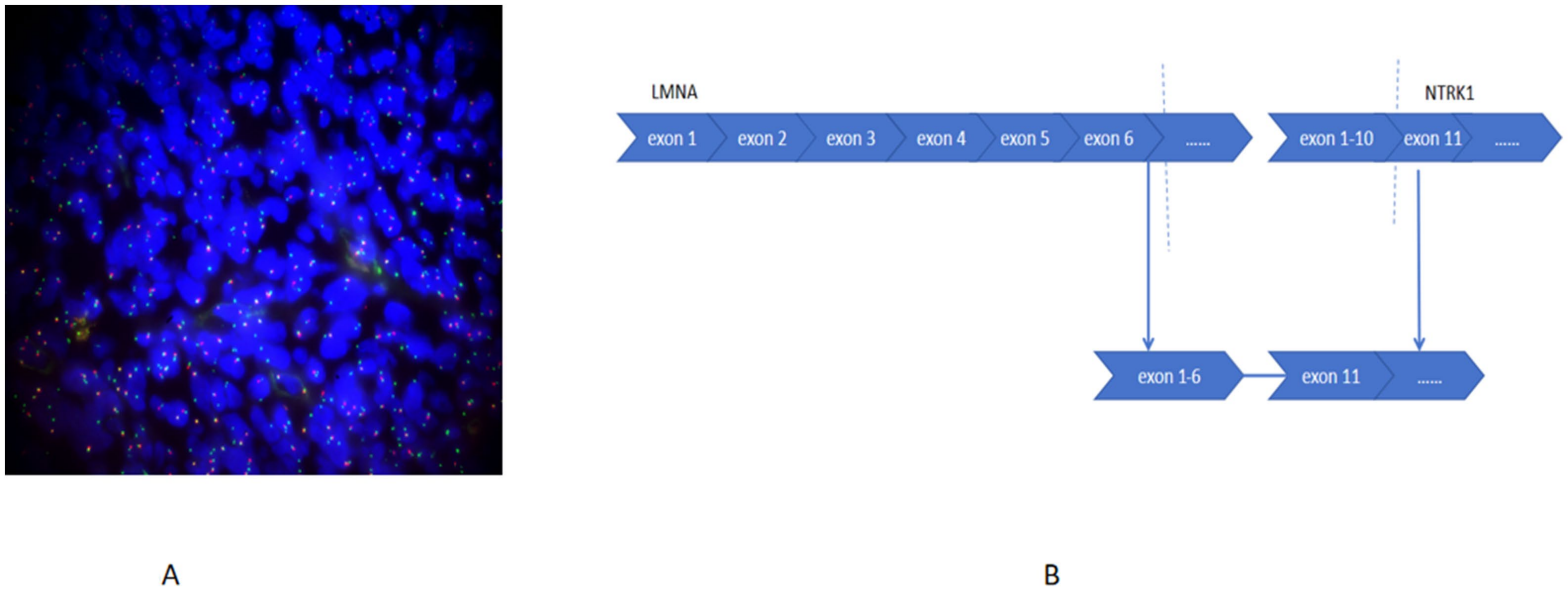
FISH analysis demonstrated the fusion of the LMNA-NTRK1 gene using an NTRK1 break-apart probe **(A)**. RNA-based sequencing identified an LMNA-NTRK1 fusion **(B)**.

Targeted RNA sequencing identified an in-frame LMNA::NTRK1 fusion, with breakpoints at LMNA exon 6 and NTRK1 exon 11 (Figures 3A,B). No other pathogenic mutations or fusions were detected.

### 2.3 First recurrence (21 months after initial resection)

The patient presented with a 2.5-cm firm nodule at the same scalp site. Gross pathology: A 2.8 cm × 2.5 cm × 2.0 cm nodule with a tan-yellow cut surface and irregular margins.

#### 2.3.1 Histopathology

**Figure 4:**
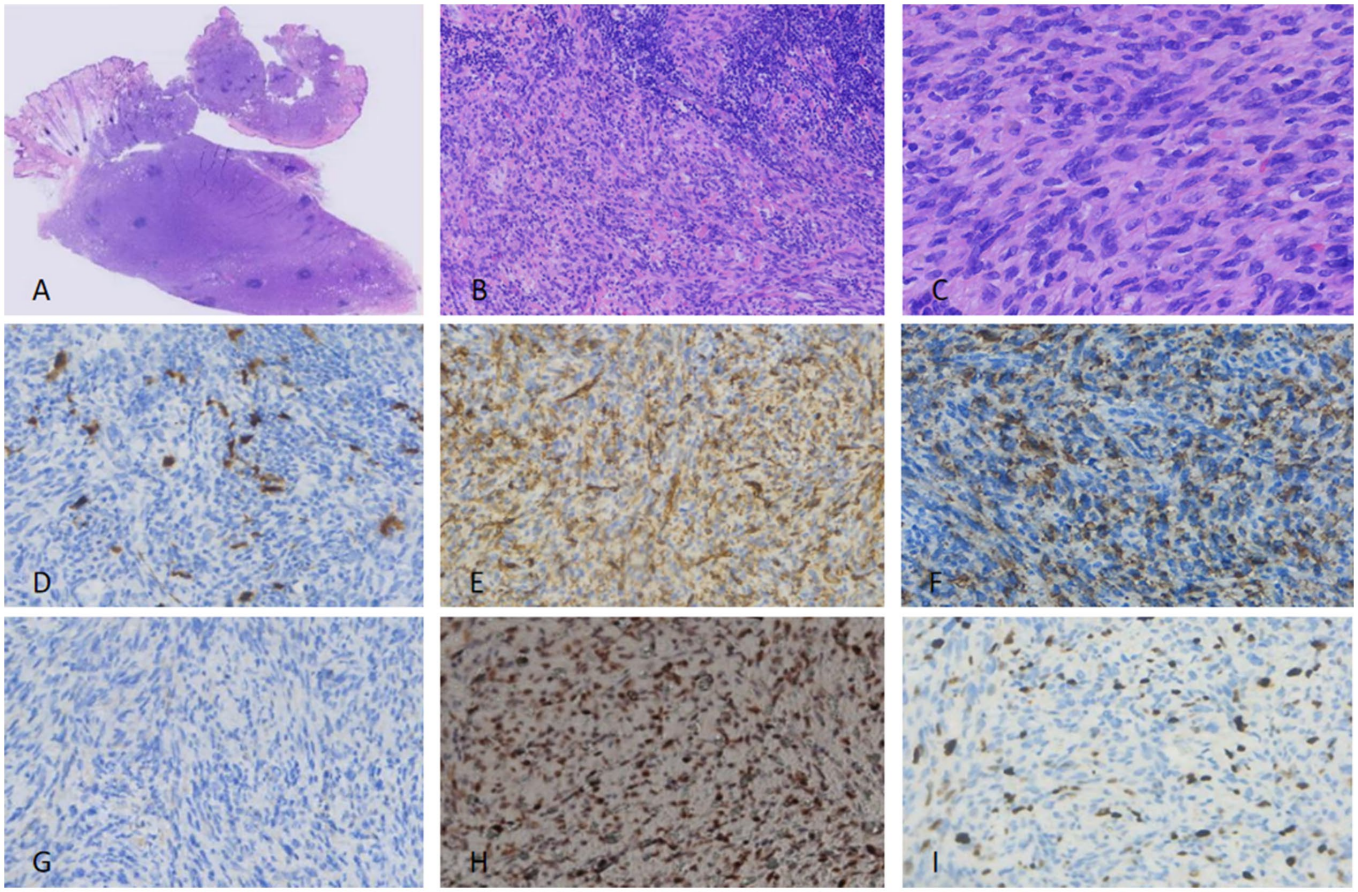
For the excised specimen of the first recurrence, tightly packed spindle-shaped cells were arranged in fascicular, whorled, and storiform patterns with lymphocyte aggregation present. Pigment cells were no longer visible. Moderate nuclear atypia and pathological mitoses were observed **(A–C)**. Immunohistochemically, the tumor cells showed hardly any positivity for S100 **(D)**—a finding distinct from the primary specimen. CD34 **(E)** and P-TRK **(F)** were focally positive, while SMA remained negative **(G)**. P53 xpression was still diffusely positive. **(H)** Ki-67 proliferation index was nearly 20% **(I)**.

Spindle cell proliferation was retained, but with increased cellular atypia, hyperchromatic nuclei, and fibrosarcoma-like malignant transformation (fascicular arrangement of pleomorphic spindle cells; Figures 4A–C). Mitotic activity was increased (3/10 HPF).

#### 2.3.2 IHC

Results were consistent with the primary tumor, except for the loss of S100 expression (Figure 4D) and an elevated Ki-67 index (20%; Figure 4I). Pan-TRK (Figure 4F), CD34 (Figure 4E), and p53 (Figure 4H) remained positive; SMA was focally positive (Figure 4G). Tumor cells were identified at the surgical margins (Figure 2B).

### 2.4 Second recurrence (6 months after first re-excision)

The patient developed a third nodule at the same site, requiring re-resection. Gross pathology: A 3.0 cm × 2.8 cm × 2.2 cm firm nodule with a grayish-tan cut surface and infiltrative margins.

#### 2.4.1 Histopathology

**Figure 5:**
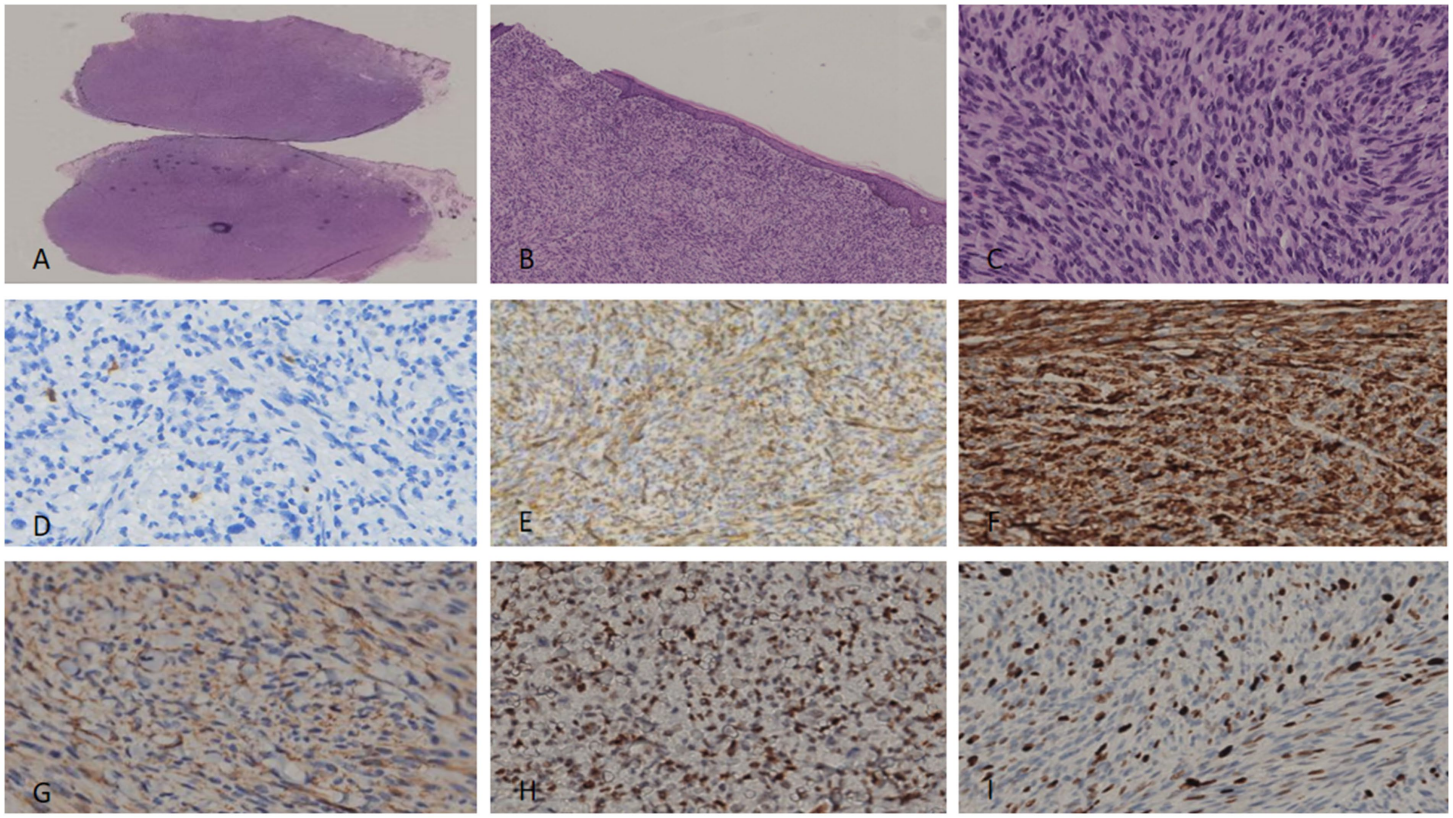
For the excised specimen of the second recurrence, tightly packed spindle-shaped cells were arranged in a storiform pattern, with obvious nuclear atypia and pathological mitoses. Pigment cells had completely disappeared **(A–C)**. Immunohistochemically, the tumor cells were negative for S100 **(D)**, while CD34 **(E)**, P-TRK **(F)**, SMA **(G)**, and P53 **(H)** showed diffuse positivity. The Ki-67 proliferation index was even higher than before, reaching more than 40% **(I)**.

Marked nuclear atypia, increased mitotic activity (5/10 HPF), and prominent fibrosarcoma-like morphology were observed (Figures 5A–C). No necrosis was identified.

#### 2.4.2 IHC

S100 remained negative (Figure 5D), while CD34 (Figure 5E), Pan-TRK (Figure 5F), SMA (Figure 5G), and p53 (Figure 5H) were diffusely positive. The Ki-67 index was 40% (Figure 5I). Surgical margins were negative for tumors (Figure 2C).

### 2.5 Treatment and follow-up

**Figure 6:**
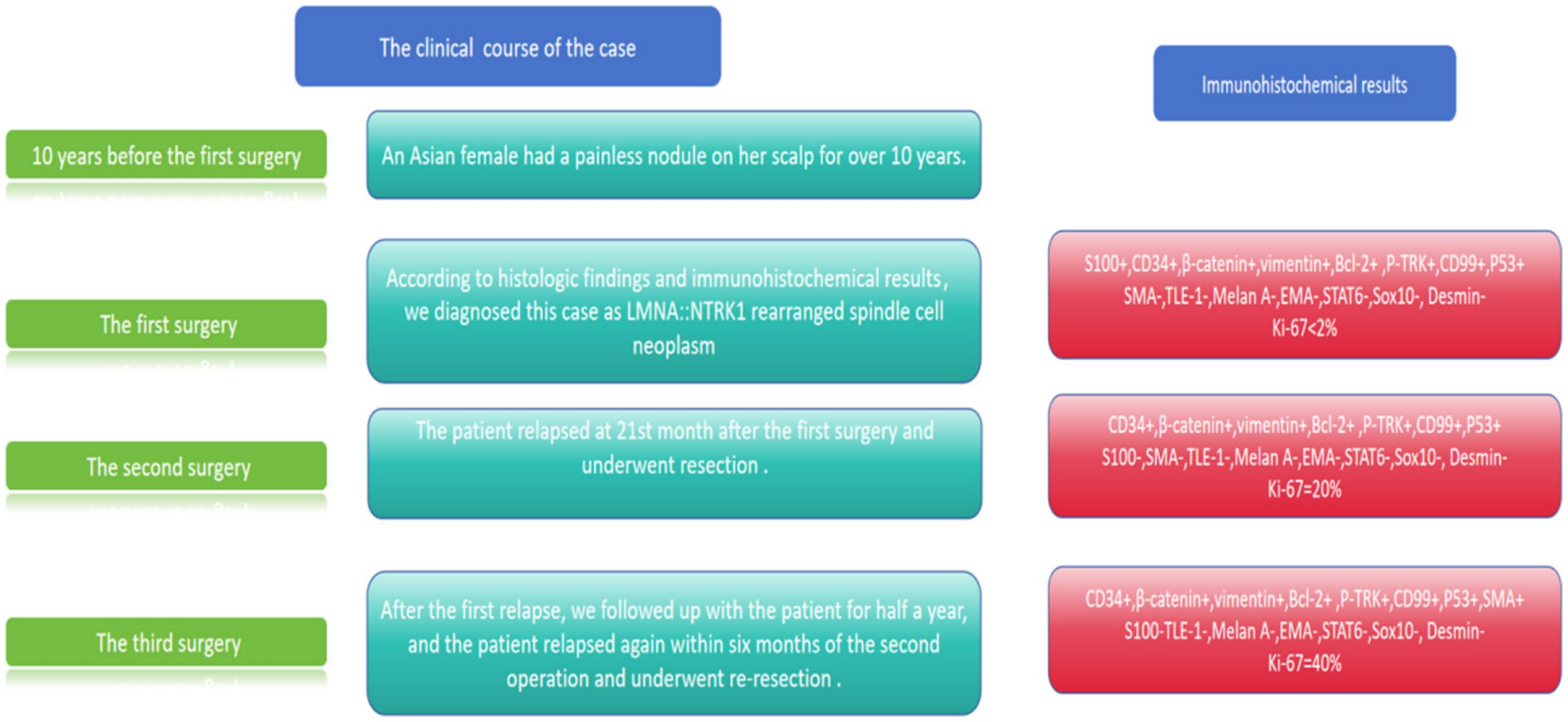
Clinical course and immunohistochemical results of our case.

The primary tumor was treated with a wide local excision (margin ≥1 cm). After the first recurrence, the patient underwent re-excision with margin control. Following the second recurrence, radical resection was performed. TRK inhibitor therapy (larotrectinib) was recommended postoperatively, but the patient declined. At 12-month follow-up after the third resection, no evidence of further recurrence or distant metastasis was detected. Clinical and pathological features are summarized in Figure 6.

## 3 Literature review

### 3.1 Search strategy

A comprehensive literature search was conducted in PubMed, Embase, and Web of Science databases using the terms (“LMNA::NTRK1” OR “LMNA-NTRK1 fusion”) AND (“spindle cell neoplasm” OR “soft tissue tumor” OR “sarcoma”) from January 2016 to March 2025. Studies were included if they reported clinical, pathological, or molecular data on LMNA::NTRK1-rearranged spindle cell neoplasms. Duplicates, review articles, and non-English studies were excluded. Two independent researchers screened titles, abstracts, and full texts, with discrepancies resolved by consensus.

### 3.2 Summary of published cases

Including our case, 40 patients with LMNA::NTRK1-rearranged spindle cell neoplasms have been reported (Table 1). Demographic features: Male-to-female ratio 19:21 (47.5%:52.5%), age range 0.2–57 years (mean ± SD 16.5 ± 17.5 years; median 19 years). Tumor locations: Limbs/trunk (25 cases, 62.5%), gastrointestinal tract (6 cases, 15%), head/neck (7 cases, 17.5%), and lungs (2 cases, 5%).

**Table 1:** Clinicopathological features of LMNA::NTRK1 spindle cell neoplasm in the literature.

Case	Author	Age/Gender	Site	Gene fusion type	Positive immunohistochemistry	Negative immunohistochemistry	Treatment	Outcomes
1	Haller et al. (4)	2 years/Female	Paravertebral lumbar	LMNA::NTRK1	α-SMA	Desmin, h-caldesmon, CD34, STAT6, EMA, CK	Excision, radiotherapy	No evidence of disease
2	Agaram et al. (5)	4 years/Female	Thigh	LMNA:NTRK1	S100, CD34, NTRK1	Sox10, HMB45, Melan A, Desmin, GFAP, STAT6	Tumor resection	No evidence of disease
3	Agaram et al. (5)	28 years/Female	Flank	LMNA::NTRK1	S100	NTRK1, Sox10, HMB45, Melan A, Desmin, GFAP, STAT6	Tumor resection	No evidence of disease
4	Agaram et al. (5)	15 years/Male	Forearm	LMNA::NTRK1	S100, NTRK1	Sox10, HMB45, Melan A, Desmin, GFAP, STAT6	NA	NA
5	Agaram et al. (5)	12 years/Male	Arm	LMNA::NTRK1	S100, CD34, NTRK1	Sox10, HMB45, Melan A, Desmin, GFAP, STAT6	Tumor resection	No evidence of disease
6	Davis et al. (6)	2 months/Female	Back	LMNA::NTRK1	S100, CD34, NTRK1, CD30	SMA	Tumor resection	No evidence of disease
7	Kohsaka et al. (7)	6 years/Female	Right elbow	LMNA::NTRK1	CD34, S100, EMA, CK	myogenic marker	Tumor resection	No evidence of disease
8	Warren et al. (8)	3 years/Female	Back	LMNA::NTRK1	S100, CD34, NTRK1	Myogenin, Desmin, CD117, DOG1, Bcl2, CD68, factor XIIIA	Tumor resection	No evidence of disease
9	Malik et al. (9)	3 years/Female	Right buttock	LMNA::NTRK1	S100, CD34, SMA, Pan-TRK	Desmin, CD117, ERG	Tumor resection	NA
10	Dupuis et al. (10)	21 years/Male	Lumber	LMNA::NTRK1	CD34,pan-TRK, S100, SMA, SATB2, H3K27me3	STAT6, DOG1, Desmin, TLE-1, Melan-A, Sox10	Targeted therapy, surgery	No evidence of disease
11	Yin et al. (11)	3 months/Female	Knee	LMNA::NTRK1	Vimentin, CD34, MSA, SMA, Pan-TRK	Alk	Chemotherapy	No recurrence
12	Yin et al. (11)	3 years/Male	Thigh	LMNA::NTRK1	Vimentin, CD34, MSA, SMA, Desmin, Pan-TRK	Alk	Chemotherapy, targeted therapy	NA
13	Kang et al. (12)	3 years/Male	Forehead	LMNA::NTRK1	Trk, S100, CD34, nestin, vimentin, CD3	CD56, SMA, Desmin, myogenin, STAT6, EMA, CK, CD1a, CD21, CD35, CD43, WT-1, MelanA, HMB45, BRAF, ALK	Tumor resection	No evidence of disease
14	So et al. (13)	40 years/Female	Calf	LMNA::NTRK1	S100, CD34, SMA, Rb, INI-1, Pan-TRK	SoxX10, STAT6, GFAP, calponin, Desmin, MUC4, CD56, CDK4, CD31, CAM5.2, MNF116, CK, EMA, p63	Tumor resection, chemotherapy	Lung metastasis
15	Atiq et al. (14)	4 years/Female	Stomach	LMNA::NTRK1	Trk, S100, CD34	Sox10, EMA Kit, DOG1, Desmin, STAT6, ALK, CK	Tumor resection	NA
16	Brčić et al. (15)	50 years/Male	Neck	LMNA::NTRK1	pan-TRK, CD34, S100	Sox10, H3K27me3	Excised with clear margins	No evidence of disease
17	Panse et al. (3)	31 years/Female	Scalp	LMNA: NTRK1	CD34, S100, pan-TRK	Sox10, CK, EMA, Desmin, CD21, CD23, ALK	Excised with clear margins	No recurrence
18	Yin et al. (26)	9 months/Male	Back	LMNA::NTRK1	S100, CD34, H3K27Me3, TRK-A, Pan-TRK	Sox-10	Tumor resection	NA
19	Yin et al. (26)	3 years/Female	Abdominal wall	LMNA::NTRK1	S100, CD34, H3K27Me3, TRK-A, Pan-TRK	Sox-10	Extended tumor resection	Local recurrence
20	Yin et al. (26)	4 years/Female	Right upper arm	LMNA::NTRK1	S100, CD34, H3K27Me3, TRK-A, Pan-TRK	Sox-10	Extended tumor resection	Local recurrence
21	Yin et al. (26)	22 years/Male	Right buttock	LMNA::NTRK1	S100, CD34, H3K27Me3, TRK-A, Pan-TRK	Sox-10	Tumor resection	No evidence of disease
22	Yin et al. (26)	23 years/Male	Rectum	LMNA::NTRK1	S100, CD34, H3K27Me3, TRK-A, Pan-TRK	Sox-10	Tumor resection	NA
23	Zhu et al. (23)	31 years/Male	Lung(Right upper lobe)	LMNA::NTRK1	S100, CD34, H3K27Me3, TRK-A, Pan-TRK	Sox-10, α-SMA, Desmin, ALK, STAT6, calponin	Tumor resection	No evidence of disease
24	Tsai et al. (20)	34 years/Male	Right lung	LMNA::NTRK1	CD34, H3K27Me3, P53, Pan-TRK	S100, P16	No resection	Alive with disease
25	Rahim et al. (18)	20 years/Female	Ileum	LMNA::NTRK1	Pan-TRK, S100, CD34, H3K27Me3	CD117, DOG1, STAT6, Sox-10, CK, EMA, Desmin, SMA, HMB-45, Melan-A, ALK1,β-calponin, SYN. CgA	Tumor resection	No recurrence
26	Tauziede-Espariat et al. (22)	21 years/Female	Thorax	LMNA::NTRK1	CD34, S100	Sox-10	NA	NA
27	Kobayashi et al. (21)	23 years/Female	Lower leg	LMNA::NTRK1	CD34, S100	STAT6	Tumor resection	Complete disease-free
28	Kobayashi et al. (21)	35 years/Male	Perineal	LMNA::NTRK1	CD34, S100	STAT6	Tumor resection	Complete disease-free
29	Czaja et al. (17)	11 years/Male	Back	LMNA::NTRK1	Pan-TRK, S100, CD34, CD30	Sox-10	Excised with clear margins	Free of disease
30	Wei et al. (16)	57 years/Male	Right buttock	LMNA::NTRK1	Pan-TRK, S100, CD34, Caldesmin	Sox-10, SMA, Desmin, EMA, ALK, STAT6, CD31, ALK	Tumor resection	No recurrence
31	Jian et al. (25)	47 years/Male	Ascending colon	LMNA::NTRK1	S100, CD34, Pan-TRK	Sox-10, CK, EMA, SMA, CD117, Desmin, DOG1, ALK	Tumor resection	No recurrence
32	Gao et al. (24)	7 years/Male	Descending colon	LMNA::NTRK1	S100, CD34, H3K27Me3, Pan-TRK	Sox-10, AE1/AE3, SMA, CD117, Desmin, DOG1, ALK, STAT6	Tumor resection	No evidence of disease
33	Gao et al. (24)	45 years/Female	Transverse colon	LMNA::NTRK1	CD34, H3K27Me3, TRK-A, Pan-TRK	S100, Sox-10, AE1/AE3, SMA, CD117, Desmin, DOG1, ALK, STAT6	Tumor resection	No evidence of disease
34	Gao et al. (24)	34 years/Female	Ascending colon	LMNA::NTRK1	CD34, H3K27Me3	Sox-10, AE1/AE3, SMA, CD117, Desmin, DOG1, ALK, STAT6	Tumor resection	No evidence of disease
35	Suurmeijer et al. (19)	4 years/Male	Mandible	LMNA::NTRK1	S100, CD34	NA	Tumor resection	No recurrence
36	Suurmeijer et al. (19)	13 years/Male	Maxilla	LMNA::NTRK1	S100, CD34	Sox-10	Tumor resection	No recurrence
37	Klubíčková N et al. (27)	3 years/Female	Lower eyelid	LMNA::NTRK1	S100, CD34, Pan-TRK	NA	Surgery, targeted therapy	Local recurrence
38	Klubíčková N et al. (27)	37 years/Female	Instep	LMNA::NTRK1	S100, CD34, Pan-TRK	NA	Tumor resection	No evidence of disease
39	Klubíčková N et al. (27)	43 years/Male	Hip	LMNA::NTRK1	S100, CD34, Pan-TRK	NA	Tumor resection	No evidence of disease

Histopathology: The majority of cases exhibited fibromatosis-like (hypocellular spindle cells in collagenous stroma) or inflammatory myofibroblastic tumor-like (fascicular growth with lymphoplasmacytic infiltration) morphology. Hemangiopericytoma-like vessels and CD34 positivity were common, mimicking SFT. Myxoid degeneration was rare. Typically, tumors showed low mitotic activity (<2/10 HPF) and minimal pleomorphism. Pigmentation and fibrosarcoma-like transformation were not reported in any prior case.

#### 3.2.1 Immunophenotype

Consistent findings included CD34 positivity (36/37 cases, 97.3%), S100 positivity (33/35 cases, 94.3%), and Pan-TRK positivity (23/23 cases, 100%). All cases were negative for Sox10 (22/22), STAT6 (17/17), DOG1 (8/8), and CD117 (7/7). SMA was positive in 60% (9/15) of cases, while Desmin was uniformly negative (19/19).

#### 3.2.2 Clinical outcomes

Follow-up data were available for 34 patients. The majority of patients (28 cases, 82.4%) remained disease-free after surgical resection. Local recurrence could occur in 3 cases (8.8%), and lung metastasis in 1 case (2.9%). Five patients (14.7%) received chemotherapy or targeted therapy. No prior cases of multiple recurrences or malignant transformations were documented.

## 4 Discussion

To our knowledge, this is the first reported case of LMNA::NTRK1-rearranged spindle cell neoplasm with three key novel features: (1) pigmentation (dendritic pigment-containing stromal cells), (2) multiple local recurrences (two episodes within 27 months), and (3) fibrosarcoma-like malignant transformation. These findings expand the morphological and clinical spectrum of LMNA::NTRK1-rearranged tumors, challenging the notion that these tumors uniformly follow an indolent course.

### 4.1 Diagnostic considerations

LMNA::NTRK1-rearranged neoplasms must be distinguished from several mimickers, particularly given their overlapping histomorphology and immunophenotype.

Pigmented dermatofibrosarcoma protuberans (DFSP): DFSP typically shows storiform spindle cell proliferation and is characterized by COL1A1: PDGFB fusion. Unlike our case, DFSP is S100-negative and STAT6-negative (29).

Melanoma often exhibits marked nuclear pleomorphism and is positive for Melan A, HMB45, and Sox10—all negative in our case (30).

ALK-rearranged melanocytic myxoid spindle cell tumor (MMySTAR): MMySTAR is characterized by myxoid stroma, melanocytic differentiation (Melan A+/HMB45+/Sox10+), and ALK fusion—all of which are features absent in our case (31).

FMR1-ALK-rearranged cutaneous myxoid spindle cell neoplasm: This entity shows myxoid stroma and whole spindle cell arrangement but lacks pigmentation and NTRK fusion (32).

PRRX1-NCOA1-rearranged fibroblastic tumor: These tumors exhibit pigmentation and S100 positivity but are Sox10-positive and harbor PRRX1-NCOA1 fusion (33, 34).

The key diagnostic triad for LMNA::NTRK1-rearranged neoplasms—CD34+, S100+, Pan-TRK + —was present in our case, confirming the diagnosis. Loss of S100 expression in recurrent lesions may represent a marker of malignant transformation. With the increasing application of bioinformatics analyses, additional biomarkers are being identified in these tumors (35, 36).

### 4.2 Mechanisms of malignant transformation

The molecular drivers of recurrence and malignant transformation in our case remain to be fully elucidated, but several factors may contribute:

p53 mutation: Diffuse p53 positivity in all tumor specimens suggests a pathogenic TP53 mutation. TP53 is a critical tumor suppressor gene; mutations are associated with increased genomic instability, malignant progression, and poor prognosis in soft tissue sarcomas (37, 38). Our findings suggest that p53 mutation may cooperate with LMNA::NTRK1 fusion to drive tumor progression, representing a potential prognostic biomarker.
Surgical margin status: Tumor cells at the margin of the first recurrence specimen may have contributed to subsequent progression, emphasizing the importance of wide local excision.
Absence of adjuvant therapy: The patient declined TRK inhibitor therapy, which may have prevented a recurrence. TRK inhibitors have been shown to induce durable responses in NTRK fusion-positive tumors, even at advanced stages (2, 28).

### 4.3 Clinical implications

Our case highlights three critical clinical implications: (1) LMNA::NTRK1-rearranged tumors may exhibit malignant transformation, requiring long-term follow-up (39), (2) p53 positivity may serve as a prognostic marker for high-risk disease, and (3) adjuvant TRK inhibitor therapy should be considered in cases with adverse features (e.g., positive margins, pleomorphism, and p53 mutation) (40). Further studies are needed to validate these findings and establish optimal treatment algorithms.

### 4.4 Limitations

This study has several limitations: (1) it is a single-case report, limiting generalizability, (2) TP53 sequencing was not performed to confirm the mutation, and (3) long-term follow-up is ongoing to assess for distant metastasis.

## 5 Conclusion

We report the first case of LMNA::NTRK1-rearranged spindle cell neoplasm with pigmentation, multiple recurrences, and fibrosarcoma-like malignant transformation. Our findings expand the morphological and clinical spectrum of this rare entity and suggest that p53 mutation may contribute to disease progression. Accurate diagnosis via integrated histopathological and molecular testing is critical, and adjuvant TRK inhibitor therapy should be considered in high-risk cases. Further collection of cases is needed to better understand the biological behavior and optimal management of LMNA::NTRK1-rearranged neoplasms.

## Data Availability

The original contributions presented in the study are included in the article/Supplementary material, further inquiries can be directed to the corresponding authors.
